# Evaluating the efficacy of switching from lamivudine plus adefovir to tenofovir disoproxil fumarate monotherapy in lamivudine-resistant stable hepatitis B patients

**DOI:** 10.1371/journal.pone.0190581

**Published:** 2018-01-12

**Authors:** Heon Ju Lee, Sang Jin Kim, Young Oh Kweon, Soo Young Park, Jeong Heo, Hyun Young Woo, Jae Seok Hwang, Woo Jin Chung, Chang Hyeong Lee, Byung Seok Kim, Jeong Ill Suh, Won Young Tak, Byoung Kuk Jang

**Affiliations:** 1 Department of Internal Medicine, Yeungnam University College of Medicine Daegu, South Korea; 2 Department of Internal Medicine, Keimyung University school of Medicine, Daegu, South Korea; 3 Department of Internal Medicine, Kyungpook National University College of Medicine, Daegu, South Korea; 4 Department of Internal Medicine, Pusan National University School of Medicine, Busan, South Korea; 5 Department of Internal Medicine, Catholic University of Daegu School of Medicine, Daegu, South Korea; 6 Department of Internal Medicine, Dongguk University College of Medicine, Gyeongju, South Korea; Universidad de Navarra, SPAIN

## Abstract

**Background:**

The efficacy of switching to tenofovir disoproxil fumarate (TDF) monotherapy from lamivudine (LAM) plus adefovir dipivoxil (ADV) combination therapy (stable switching) in patients with LAM-resistant chronic hepatitis B (CHB) and undetectable hepatitis B virus (HBV) DNA is not clear.

**Methods:**

In this non-inferiority trial, patients with LAM-resistant CHB and undetectable serum HBV DNA (<20 IU/mL) for >6 months after initiating LAM+ADV combination therapy were randomized (1:2) either to continue the combination therapy (LAM+ADV group, n = 58) or switched to TDF monotherapy (TDF group, n = 111). They were followed-up with serum biochemistry tests and HBV DNA measurement at 12-week intervals for 96 weeks. The primary endpoint of this study was the proportion of patients with viral reactivation at week 96.

**Results:**

Patients with CHB enrolled in this study (n = 169) included 74 patients with compensated liver cirrhosis. In total, 9 patients (4 in the LAM+ADV group and 5 in the TDF group) dropped-out from the study. After a mean follow-up period of 96 weeks, the proportion of HBV reactivation observed was 6.8% (4/58) in the LAM+ADV group and 4.5% (5/111) in the TDF group by using intention-to-treat analysis (difference, -2.3%; 95% CI, -9.84–5.24%). None of the subjects in either group experienced viral reactivation based on per protocol analysis. No serious adverse reactions were observed. In the subgroup analysis for estimated glomerular filtration rate (eGFR) before and after treatment, decreased eGFR was observed only in the TDF group with cirrhosis (85.22 vs. 79.83 mL/min/1.73 m^2^, p = 0.000)

**Conclusions:**

Stable switching to TDF monotherapy yielded non-inferior results at 96 weeks compared to the results obtained with LAM+ADV combination therapy in patients with LAM-resistant CHB and undetectable HBV DNA. However, TDF monotherapy in patients with cirrhosis requires close attention with respect to renal function.

**Trial registration:**

ClinicalTrials.gov NCT01732367

## Introduction

The goal of chronic hepatitis B (CHB) therapy is to improve the quality of life and duration of survival by preventing the progression of the disease to hepatocellular carcinoma (HCC) and death. This can be achieved by suppressing the replication of hepatitis B virus (HBV). A reduction in the histological activity of HBV decreases the risk of cirrhosis and HCC [1]. However, chronic HBV infection cannot be completely eradicated owing to the persistence of covalently closed circular DNA in the nucleus of the infected hepatocytes, responsible for HBV reactivation [2, 3]. Lamivudine (LAM), a nucleoside analog, was the first oral antiviral agent used as a standard treatment for CHB. However, LAM is associated with a high incidence of resistance, and the cumulative rate increases up to 70% at 5 years [4–6].

In patients with LAM resistance, combination treatment with adefovir dipivoxil (ADV) and LAM did not show higher antiviral efficacy than ADV monotherapy. However, development of ADV resistance was significantly lower with the combination therapy than with ADV monotherapy [7, 8]. Therefore, LAM+ADV combination therapy was considered as standard therapy for patients with LAM resistance before tenofovir disoproxil fumarate (TDF) therapy was introduced [9]. Moreover, LAM+ADV therapy needs to be continued even after the complete suppression of HBV DNA.

TDF is an oral nucleotide analog, which was approved in 2008. It exhibits sustained antiviral effects in patients who had been exposed to other antiviral agents as well as in those who received initial treatment, without the development of genotypic resistance [10]. In patients with LAM-resistant CHB, TDF showed good antiviral effects without any significant adverse events [11]. Recently published guidelines recommend TDF or entecavir as the first-line treatment in naïve CHB patients [6, 12, 13]. When patients with LAM resistance were treated either with TDF alone or in combination with emtricitabine, no significant difference was observed in the proportion of patients with HBV DNA <69 IU/mL (89.4 vs. 86.4%) [14], indicating that TDF monotherapy does not affect the virus suppression rate and prevent novel viral mutations. A recent study showed that treatment of CHB with TDF monotherapy for 8 years maintained effective viral suppression with no evidence of TDF resistance [15]. Recently, the efficacy of TDF monotherapy was reported to be comparable to that of combination therapy with TDF and ETV at 96 weeks [16].

Although prospective cohort studies comparing TDF monotherapy and LAM+ADV combination therapy are lacking, many guidelines [6, 12, 17] recommend both LAM+ADV and TDF monotherapy as standard treatments for LAM-resistant CHB. A recent guideline of the American Association for the Study of Liver Diseases [13] recommended TDF monotherapy or adding TDF to existing standard therapy based on the results of a previous study [14]. If TDF treatment for LAM-resistant CHB patients was less effective than LAM+ADV treatment, these patients should not be switched to TDF monotherapy. When LAM-resistant patients, who had already received LAM and ADV combination therapy, whose HBV DNA negativity was well maintained, and who were not ADV resistant, were switched to TDF monotherapy, they were theoretically likely to maintain their virus response. However, there are no reliable evidences to substantiate these hypotheses.

In this study, we evaluated the efficacy of stable switching from LAM+ADV combination therapy to TDF monotherapy in patients with LAM-resistant CHB and undetectable HBV DNA at 96 weeks.

## Methods

### Study design

This study was a multicenter prospective randomized open-label 96-week trial conducted in patients with LAM-resistant CHB and undetectable serum HBV DNA (<20 IU/mL) for more than 6 months after the initiation of LAM+ADV combination therapy. The sample size was calculated assuming a non-inferiority design. The primary endpoint of this study was the proportion of patients without viral reactivation at week 96. The expected proportion in the LAM+ADV combination therapy was set at 98%. The non-inferiority margin of the difference in the proportions between the two groups was considered 6%. In order to have 80% power and 5% type I error with 1:2 (LAM+ADV:TDF) allocation, and considering a 10% drop-out rate, a total of 171 patients (57 for LAM+ADV arm, 114 for TDF arm) were screened between August 2013 and February 2015 across 6 centers in Korea, of which 2 patients were excluded. Finally, 169 patients were enrolled in this study.

The patients were randomized to the two arms using a centralized procedure and an interactive web response system to indicate continuation of LAM+ADV combination therapy (LAM+ADV group) or a switch to TDF monotherapy (TDF group). The inclusion criteria were male and female adult (age >18 years) patients with CHB in the presence or absence of compensated liver cirrhosis (Child–Pugh class A). Liver cirrhosis was categorized based on typical image data, including nodular liver surface, splenomegaly, and the presence of intra-abdominal collateral vessels, which indicate increased portal venous pressure. Patients with positive serum hepatitis B surface antigen (HBsAg) for at least 6 months were enrolled if they had undetectable serum HBV DNA (<20 IU/mL) at screening and were receiving LAM+ADV. All patients were confirmed to exhibit genotypic resistance to LAM. Patients with decompensated liver cirrhosis or HCC at the screening stage were excluded. Other exclusion criteria were as follows: virus co-infections by hepatitis C virus, hepatitis D virus, or HIV; elevated alanine aminotransferase (ALT, >2 times the normal upper limit); elevated creatinine (>2 mg/dL); pregnant or lactating women; women planning for pregnancy in the following 3 disorders; uncontrolled severe concomitant diseases such as severe cardiovascular diseases and other infections; and subjects lacking capability to understand and provide informed consent. Written informed consent was obtained from all study participants. This study was approved by the Keimyung University Dongsan Medical Center Institutional Review Board (IRB) and IRBs of each investigational site, and it was registered with ClinicalTrials.gov (NCT01732367).

### End points and monitoring

The primary efficacy endpoint was the proportion of patients with viral reactivation at week 96. The secondary endpoints were based on the proportion of patients with viral reactivation at week 48, drug-resistant mutations at weeks 48 or 96 during the randomized therapy, biochemical breakthrough at weeks 48 or 96, unusual serologic response, and patient safety. At each visit every 3 months, routine liver biochemistry, hepatitis B serology (presence of HBeAg, HBe antibody, HBsAg and HBs antibody), and serum HBV DNA measurements were performed. HBV DNA was measured using m2000 Real-Time System (Abbott Molecular Inc., Des Plaines, IL, USA) with a lower limit of detection of 20 IU/mL. Mutational analysis to determine the presence of drug-resistant mutations was performed at the time of virus reactivation. Genotypic resistance to LAM was tested by restriction fragment mass polymorphism (RFMP; Genematrix, Seongnam-si, Gyeonggi, Korea assay).

At the time of enrollment, all patients had undetectable serum HBV DNA levels (<20 IU/mL). If there was evidence of viral reactivation with TDF therapy, LAM was added to the treatment according to the protocol. The criterion for virus reactivation was defined as HBV DNA levels >40 IU/mL at 2 consecutive time-points, or persistent HBV DNA levels of 20–40 IU/mL at 3 consecutive time-points.

### Statistical analysis

All statistical analyses were performed using SPSS v. 22.0 software (Chicago, IL, USA). The chi-square test was used to compare proportions for categorical variables and Fisher’s exact test was used to assess statistical significance. Between-group comparison of continuous variables with normal distribution was conducted using Student’s t test. Continuous variables with skewed distribution were expressed as median values with 1^st^ and 3^rd^ interquartile ranges (IQR) and analyzed using the Mann–Whitney test. p <0.05 was considered statistically significant. If the mean was less than twice the standard deviation, such as the mean values of ALT and APRI, the median and IQRs were used to analyze non-normal distributions.

## Results

### Baseline characteristics

Patients with CHB (n = 169), including 74 with compensated cirrhosis and receiving LAM+ADV combination therapy for >6 months, were enrolled in this study. They were randomized to receive either TDF monotherapy (TDF group, n = 111) or to continue LAM+ADV combination therapy (LAM+ADV group, n = 58). The baseline demographic and laboratory data are summarized in Table 1.

**Table 1:** Baseline characteristics of patients.

	Total (n = 169)	LAM+ADV (n = 58)	TDF (n = 111)
Age (year); mean ± SD	52.02 ± 10.39	49.47 ± 10.87	53.35 ± 9.91
Gender (Male); n (%)	109(64.5)	38 (65.5)	71 (64.0)
Weight, Kg; mean ± SD	64.32 ± 10.99	65.06 ± 10.31	63.94 ± 11.35
ALT, IU/L; median (IQR)	20 (16–28)	18 (15–25)	21 (16–28)
normal ALT (<45 IU/L), n (%)	160(94.6)	56(96.6)	104(93.7)
Bilirubin, mg/dL; mean ± SD	0.83 ± 0.39	0.80 ± 0.39	0.84 ± 0.39
Albumin, mg/dL; mean ± SD	4.60 ± 0.33	4.57 ± 0.35	4.61 ± 0.32
Cr, mg/dL; mean ± SD	0.89 ± 0.18	0.87 ± 0.18	0.89 ± 0.18
eGFR (mL/min/1.73 m^2^)	90.09 ± 17.81	94.33 ± 19.79	87.93 ± 16.39
INR; mean ± SD	1.05 ± 0.11	1.02 ± 0.15	1.06 ± 0.08
HBeAg (+), n (%)	29 (17.2)	12 (20.7)	17 (15.3)
Duration of undetectable HBV DNA before enrolled (weeks); median (IQR)	160.9 (109.9–202.3)	176 (120.4–124.0)	152.1 (109.8–200.0)
APRI; median (IQR)	0.35 (0.25–0.52)	0.31 (0.23–0.51)	0.36 (0.27–0.54)
Cirrhosis, n (%)	74 (43.8%)	18 (31.0%)	56 (50.5%)

ADV, adefovir dipivoxil; ALT, alanine aminotransferase; Cr, creatinine; eGFR, estimated glomerular filtration rate; HBeAg, hepatitis B envelope antigen, HBV, hepatitis B virus; INR, international normalized ratio; LAM, lamivudine; TDF, tenofovir disoproxil fumarate; IQR, interquartile range; APRI, AST to platelet ratio index.

**Fig 1:**
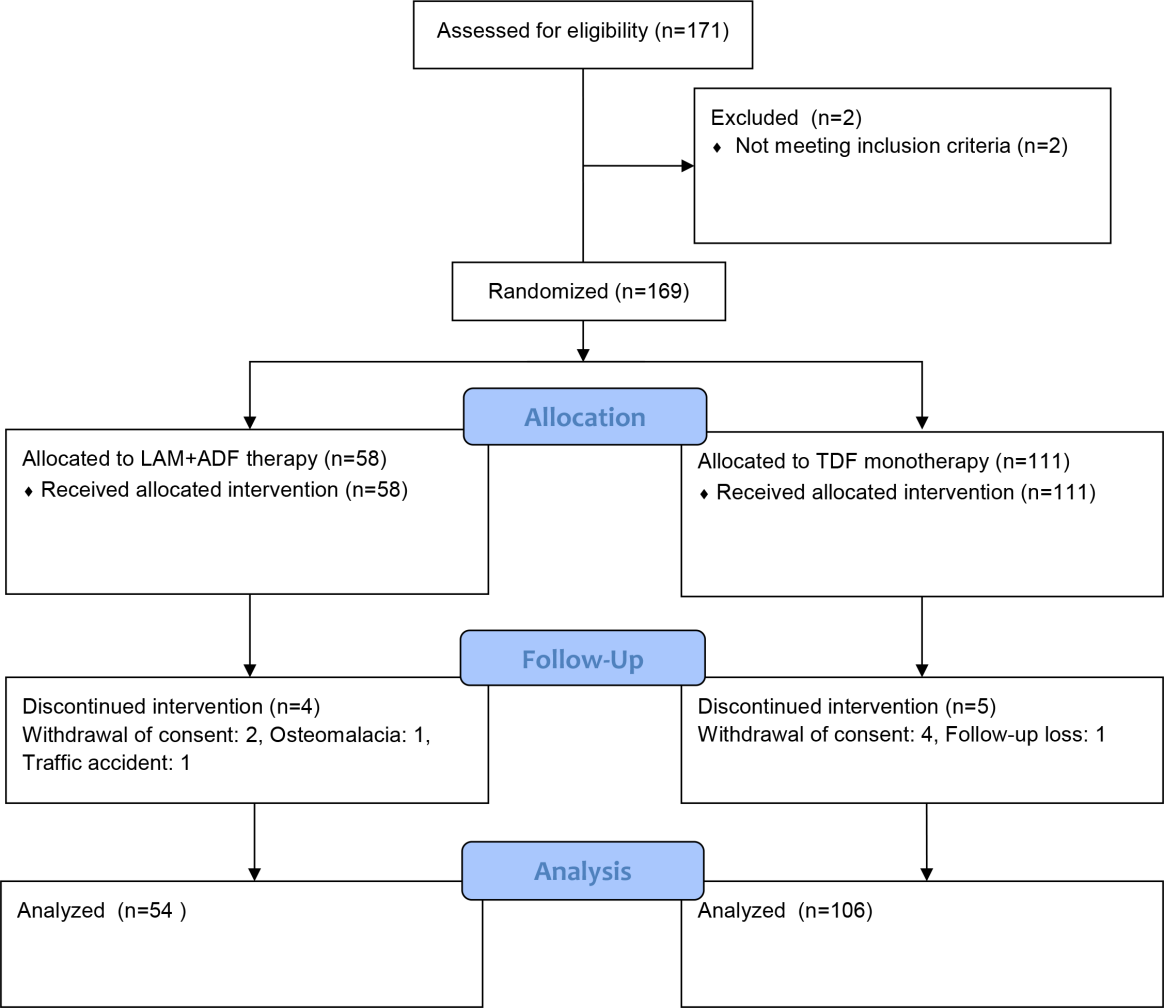
Study design and patient flow for both arms.

The patient flow is summarized in Fig 1. The patients received LAM for a median of 36 months (10–120) prior to the addition of ADV. After introduction of ADV, the median duration of LAM+ADV therapy was 56.5 months (7–109). Resistance mutation was confirmed in all patients. In this study, mutations were found at positions rt180, rt204, and rt180+rt204 in 110 patients. The presence of mutation in other patients was confirmed by primary care physicians; they were then referred to the university hospital. The profiles of resistance mutation are presented in Table 2.

**Table 2:** LAM-resistance mutation profiles and previous treatment period.

	Total (n = 169)	LAM+ADV (n = 58)	TDF (n = 111)	p value
Resistance mutations of HBV, n (%)				
rt180	73 (43.2)	21 (36.2)	52 (46.8)	0.139
rt204	106 (62.7)	35 (60.3)	71 (64.0)	0.210
rt180+rt204	69 (40.8)	18 (24.0)	51 (45.9)	0.150
Missing	53 (31.4)	17 (29.3)	36 (32.4)	0.678
Duration of LAM Tx (month); median (range)	36 (10–120) (n = 138)	33 (10–88) (n = 48)	39 (13–120) (n = 90)	0.095
Duration of LAM+ADV Tx (month); median (range)	56.50 (7–109) (n = 166)	59 (8–108) (n = 56)	55 (7–109) (n = 110)	0.053

IQR, interquartile range; ADV, adefovir dipivoxil; HBV, hepatitis B virus; LAM, lamivudine; TDF, tenofovir disoproxil fumarate.

### Comparison of virus reactivation between the LAM+ADV combination group and the TDF group at week 96

Before the end of the study, 9 patients (4 in the LAM+ADV group and 5 in the TDF group) withdrew from the study. LAM+ADV treatment was discontinued in 4 patients owing to the withdrawal of consent (n = 2), traffic accident (n = 1), and osteomalacia (n = 1). TDF was discontinued in 5 patients owing to withdrawal of consent (n = 4) and follow-up loss after occurrence of HCC (n = 1). After a mean follow-up period of 96 weeks, the proportion of HBV reactivation was 6.8% (4/58) in the LAM+/ADV group and 4.5% (5/111) in the TDF group by using intention-to-treat analysis (difference, -2.3%; 95% CI, -9.84–5.24%). When this result was analyzed per protocol, none of the subjects experienced viral reactivation in either of the groups. Switching to TDF was non-inferior at 96 weeks compared to LAM+ADV therapy. Although transient virologic rebound occurred in 16 patients (7 in the LAM+ADV group and 9 in the TDF group) through 96 weeks, HBV DNA was undetectable in all patients at the next visit (Table 3).

**Table 3:** Details of the 25 patients with transient virologic rebound or withdrawal from the trial.

No	Group	1 month	3 months	6 months	9 months	12 months	15 months	18 months	21 months	24 months	f/u
1	LAM+ADV								262		
2	LAM+ADV			48							
3	LAM+ADV	23				21					
4	LAM+ADV				1,080				43.6		
5	LAM+ADV				61.2						
6	LAM+ADV	25.8				20			20		
7	LAM+ADV	463									
8	LAM+ADV		withdrew	N/A	N/A	N/A	N/A	N/A	N/A	N/A	N/A
9	LAM+ADV					withdrew	N/A	N/A	N/A	N/A	N/A
10	LAM+ADV	withdrew	N/A	N/A	N/A	N/A	N/A	N/A	N/A	N/A	N/A
11	LAM+ADV		withdrew	N/A	N/A	N/A	N/A	N/A	N/A	N/A	N/A
12	TDF			26							
13	TDF									132	<20
14	TDF	32									
15	TDF					22					
16	TDF		13,400								
17	TDF									35.4	<20
18	TDF	98.2									
19	TDF	30.3									
20	TDF	1590									
21	TDF					withdrew	N/A	N/A	N/A	N/A	N/A
22	TDF				withdrew	N/A	N/A	N/A	N/A	N/A	N/A
23	TDF		withdrew	N/A	N/A	N/A	N/A	N/A	N/A	N/A	N/A
24	TDF		withdrew	N/A	N/A	N/A	N/A	N/A	N/A	N/A	N/A
25	TDF		withdrew	N/A	N/A	N/A	N/A	N/A	N/A	N/A	N/A

Each value was IU; N/A: not available due to withdrawal from study, Blank: Undetectable HBV DNA. ADV, adefovir dipivoxil; f/u, Follow-up; LAM, lamivudine; TDF, tenofovir disoproxil fumarate.

### Serological and biochemical responses

HBsAg seroconversion was observed in 1 patient in the TDF group (1/106, 0.94%). The number of patients with HBeAg loss at week 96 was 4/12 (33.3%) and 5/17 (29.4%) in the LAM+ADV and TDF groups, respectively. HBeAg seroconversion was observed in 2 patients (2/17, 11.7%) in the TDF group. ALT normalization was observed in 1 patient in the LAM+ADV group and 4 patients in the TDF group (p = 0.67). No significant differences were observed in levels of serum bilirubin, albumin, creatinine, and INR. The outcomes at week 96 are summarized in Table 4.

**Table 4:** Comparison between two groups after 96-week follow-up.

	Total (n = 160)	LAM+ADV (n = 54)	TDF (n = 106)	p value
HBV reactivation(ITT)	9/169	4/58(6.8%)	5/111(4.5%)	0.51
HBV reactivation(PP)	0/160	0/54	0/106	
ALT, IU/L; median (IQR)	21(17–29)	19(14–27)	22(18–30)	0.023
ALT normal (<45 IU/L), n (%)	146(91.3)	50(92.6)	96(90.6)	0.774
ALT normalization, n/N (%)	5/8(62.5)	1/2(50)	4/6(66.6)	0.67
Bilirubin, mg/dL; mean ± SD	0.79±0.35	0.76±0.33	0.80±0.36	0.497
Albumin, mg/dL; mean ± SD	4.61±0.30	4.57±0.30	4.62±0.29	0.279
Cr, mg/dL; mean ± SD	0.92±0.19	0.88±0.20	0.93±0.18	0.081
eGFR (mL/min/1.73 m^2^); mean ± SD	87.75±20.21	94.17±23.35	84.47±17.63	0.003
INR; mean ± SD	1.03±0.09	1.02±0.11	1.03±0.83	0.083
HBeAg loss, n/N (%)	9/29(31.0)	4/12(33.3)	5/17(29.4)	0.82
HBeAg seroconversion, n/N (%)	2/29(6.8)	0/12(0)	2/17(11.7)	0.22
HBeAg reversion, n (%)	1/131(0.7)	0/42(0)	1/89(0.2)	0.49
HBsAg seroconversion, n/N (%)	1/160(0.6)	0/54(0)	1/106(0.9)	1

ADV, adefovir dipivoxil; ALT, alanine aminotransferase; Cr, creatinine; eGFR, estimated glomerular filtration rate; HBeAg, hepatitis B envelope antigen; HBsAg, hepatitis B surface antigen; HBV, hepatitis B virus; INR, international normalized ratio; ITT, intention-to-treat; HCC, hepatocellular carcinoma; LAM, lamivudine; PP, per-protocol; TDF, tenofovir disoproxil fumarate; IQR, interquartile range.

### Safety

No serious adverse events were identified to be associated with administration of the study drug. HCC occurred in 2 patients in the TDF group throughout the 96 weeks of treatment. However, there was no significant difference between the treatment groups (p = 1.0). The safety profiles are described in Table 5. Serum phosphate levels <2 mg/dL were observed in 6 and 4 patients in the LAM+ADV and TDF groups, respectively (p = 0.124). Moreover, 1 patient developed osteomalacia at 9 months in the LAM+ADV group (p = 0.17). None of the patients experienced serum creatinine increase >0.5 mg/dL from the baseline value. The eGFR, which was calculated by the Modification of Diet in Renal Disease study equation, exhibited a value < 50 mL/min/1.73 m^2^ at week 96 in 1 patient in the TDF group. However, no decrease in eGFR in the LAM+ADV group was observed (p = 0.47). The patient who presented with decreased eGFR at week 96 in the TDF group showed improved eGFR (>50 mL/min) at the next visit after week 96. The eGFR between the baseline and at week 96 in the LAM+ADV group did not differ significantly (94.33 vs. 94.17 mL/min/1.73 m^2^, p = 0.935); however, it significantly decreased in the TDF group (87.93 vs. 84.47 mL/min/1.73 m^2^, p = 0.008). In the subgroup analysis, the patients in each group were divided into cirrhosis and non-cirrhosis subgroups. The eGFR of the non-cirrhotic group between baseline and at week 96 did not differ significantly (93.80 vs. 92.86 mL/min/1.73 m^2^, p = 0.555). However, it significantly decreased in the cirrhosis group (85.44 vs. 81.33 mL/min/1.73 m^2^, p = 0.008). For the cirrhosis subgroup, the eGFR between the baseline and at week 96 and treated with LAM+ADV group did not differ significantly (86.09 vs. 85.78 mL/min/1.73 m^2^, p = 0.931); decreased eGFR was observed only in the TDF group with cirrhosis (85.22 vs. 79.83 mL/min/1.73 m^2^, p = 0.000). Despite no significant difference between the baseline creatinine in both groups, the eGFR in the TDF group at baseline was significant lower than that in the LAM+ADV group.

**Table 5:** Safety profiles at 96 weeks.

	Total (n = 169)	LAM+ADV (n = 58)	TDF (n = 111)	p value
Serious adverse events	9(5.33%)^a^	4(6.90%)	5(4.50%)	1
Grade 3 or 4 adverse events	0	0	0	N/A
Discontinuation d/t adverse events	1(0.59%)^b^	1(1.72%)^b^	0	0.354
Dose reduction d/t adverse events	1(0.59%)^c^	1(1.72%)	0	0.354
ALT flare^d^	0	0	0	N/A
Hepatocellular carcinoma	2(1.18%)^e^	0	2(1.80%)^e^	1
Deaths	1(0.59%)^f^	1(1.72%)^f^	0	0.354
Serum Cr ≥ 0.5 mg/dL above baseline	0	0	0	N/A
eGFR < 50 mL/min/1.73 m^2^ at week 96	1(0.59%)	0	1(0.90%)	0.47
Serum Phosphate <2.0 mg/dL	10(5.92%)^g^	6(10.34%)	4(3.6%)	0.124

^a^ LAM+ADV group: Pedestrian traffic accident, diabetes mellitus, senile cataract, hydrocele; TDF group: cholecystitis, trans-arterial chemoembolization, esophageal bleeding, enteritis, bladder cancer. None of these was identified to be associated with the study drug administration

^b^ LAM+ADV group: osteomalacia at 9 months

^c^ ADV dosage was reduced after 3 months owing to mild decrease Grade 2 in GFR (50.9 mL/min/1.73 m^2^)

^d^ ALT flare was defined as the increase of ALT > 5 times the normal upper limit.

^e^ HCC was diagnosed at 9 months and treated using surgical resection at another hospital and follow-up loss; other 1 patient was diagnosed at 18 months and treated using trans-arterial chemoembolization

^f^ Pedestrian traffic accident: A 32-year-old man without liver cirrhosis died in a car accident while walking on a road.

^g^ Observed without significant symptoms and dose reduction

ADV, adefovir dipivoxil; ALT, alanine aminotransferase; Cr, creatinine; eGFR, estimated glomerular filtration rate; LAM, lamivudine; TDF, tenofovir disoproxil fumarate.

## Discussion

In this study, we showed that stable switching from LAM+ADV to TDF was safe and effective in patients with LAM-resistant CHB and undetectable HBV DNA. At week 96, none of the subjects in either group experienced viral reactivation. Although transient virologic rebound occurred in several patients in both groups throughout 96 weeks, HBV DNA was undetectable in all patients at the next visit.

Recently, it was reported that switching to TDF monotherapy shows superior viral suppression in patients who exhibited a suboptimal response to LAM+ADV treatment [18, 19]. However, these retrospective studies evaluated the effect of TDF monotherapy as a rescue therapy for patients with suboptimal response to LAM+ADV. There is insufficient evidence on the efficacy of stable switching from LAM+ADV treatment to TDF monotherapy. Although, TDF monotherapy demonstrated long-term viral suppression, it has not been compared with LAM+ADV therapy [20]. This is the first prospective randomized control study on stable switching to TDF monotherapy from LAM+ADV combination therapy in patients with LAM-resistant CHB and undetectable HBV DNA. The results of this study validate our hypothesis that TDF monotherapy can be as effective as LAM+ADV combination therapy in patients with stable LAM-resistant CHB.

The current guidelines do not clearly suggest a definite termination point for antiviral therapy in CHB. Goals of antiviral treatment are achievement of a sustained suppression of HBV replication and a decrease in morbidity and mortality related to CHB. This suggests that life-long antiviral therapy is necessary for patients with CHB. However, in many patients with CHB, antiviral treatment fails to maintain effective viral suppression owing to various reasons. Poor adherence to pharmacotherapy is an important factor that contributes to the development of a high rate of viral breakthrough. In a recent retrospective study of 7381 US Medicaid patients receiving treatment for HIV infection, individuals who received antiretroviral therapy in a single-tablet regimen (n = 1797) were significantly more likely to adhere (≥95%) to the therapy than patients who were treated with ≥2 tablets per day (n = 5584) [21]. In reality, several patients with CHB discontinue their antiviral treatment owing to various reasons, including lack of awareness about disease severity and the complexity of treatment regimens, such as HIV treatment protocols. Therefore, antiviral treatment as a single-tablet regimen would be more effective in increasing adherence in patients with CHB, similar to that observed in patients with HIV. In addition, despite differences in cost among countries, switching from LAM+ADV combination therapy can effectively lower the cost of antiviral treatment. In South Korea, LAM(Zeffix®)+ADV(Hepsera®) combination therapy ($148 per month) is more expensive than TDF(Viread®) therapy ($130 per month) when innovator drugs are prescribed.

To date, various adverse effects of TDF therapy such as renal dysfunction, hypophosphatemia, and fracture have been reported. A recent guideline of the American Association for the Study of Liver Diseases recommends the annual evaluation of creatinine clearance in patients treated with both ADV and TDF owing to potential renal impairment by both nucleotides. Moreover, a recent meta-analysis evaluating renal function in CHB showed that TDF monotherapy and LAM+ADV combination therapy reduced the eGFR (TDF: -9.53, CI: -14.31 to -4.89 vs. LAM+ADV: -0.39, CI: -42.48 to 41.21) [22]. The risk of renal and adverse bone effects was generally low (<2%). However, a systematic review and meta-analysis of 17 randomized control trials revealed no increased risk of severe proteinuria, hypophosphatemia, or fractures associated with TDF therapy in patients with HIV infection [23].

The eGFR at week 96 decreased significantly from the baseline only in the TDF group. According to the subgroup analysis, there was no statistically significant difference between the TDF group and the LAM + ADV group before or after treatment in the subgroup without cirrhosis. In the cirrhosis subgroup, the eGFR in the LAM + ADV group with cirrhosis did not decrease significantly after treatment, whereas the eGFR in the TDF group with cirrhosis decreased significantly after treatment with TDF. These results indicate that the high proportion of patients with cirrhosis decreased the eGFR in the TDF group. Additional studies are required in this regard, and the use of TDF in patients with cirrhosis requires close attention. The occurrence of HCC is the most serious complication in patients with CHB in the presence or absence of cirrhosis. In this study, HCC occurred in 2 patients in the TDF group. Both patients had liver cirrhosis before enrollment to this clinical trial. As mentioned previously, the TDF group included a larger number of patients with cirrhosis and older patients than the LAM+ADV group. Therefore, these factors might have influenced the occurrence of HCC. In addition, there was no statistically significant difference in HCC incidence between the two groups.

This study has several limitations. First, some baseline characteristics, such as the proportion of patients with cirrhosis and mean age, were different between the 2 groups. Despite randomization, these baseline characteristics might have influenced several adverse effects, such as eGFR impairment and occurrence of HCC. Second, bone mineral density was not evaluated in the patients who participated in this study; osteoporosis or osteopenia is a well-known adverse effect of TDF therapy. However, many studies have already shown that TDF does not significantly affect bone mineral density when compared to other antiviral agents [24]. Third, some patients were transferred from private clinics for enrollment in this study and some of those individuals did not have a precise profile for LAM mutation and LAM or LAM+ADV therapy duration. However, there is no doubt that all patients had YMDD mutants because if they did not have medical records regarding the presence of YMDD mutation, the National Health Insurance Service would not have covered the cost of the LAM+ADV rescue therapy. Another limitation is that for the evaluation of renal function, eGFR was calculated based on serum creatinine; however, urine proteinuria or urine beta-2 microglobulin was not determined.

In conclusion, we showed that switching to TDF monotherapy yields non-inferior results at 96 weeks compared to that with continuation of LAM+ADV combination therapy in patients with LAM-resistant CHB and undetectable HBV DNA. Therefore, stable switching to TDF monotherapy serves to increase medication adherence in patients with LAM-resistant CHB undergoing combination therapy with LAM and ADV. However, TDF monotherapy in patients with cirrhosis requires close attention with respect to renal function.

## Supporting information

S1 Checklist Consort checklist for this study. (DOC)

S1 Protocol Protocol of this study. (DOC)

S1 Dataset These are the data of all about this study. (XLS)
