# Supplementary material for: Evaluating the efficacy of switching from lamivudine plus adefovir to tenofovir disoproxil fumarate monotherapy in lamivudine-resistant stable hepatitis B patients
Source: PLoS One. 2018 Jan 12;13(1):e0190581. doi: 10.1371/journal.pone.0190581 (PMC5766122; doi:10.1371/journal.pone.0190581)
Supplement: S1 Protocol — (DOC) [file pone.0190581.s002.doc]

**Clinical trial plan summary**

| **Title** | Randomized Trial of Tenofovir versus Lamivudine plus Adefovir in Lamivudine plus Adefovir Treated Lamivudine-resistant chronic hepatitis B patients with Undetectable Hepatitis B Virus DNA |
| --- | --- |
| **Subjects** | This study’s subjects are hepatitis B patients with Lamivudine resistance who were treated with combination therapy of Lamivudine and Adefovir and maintained HBV DNA < 51 copies/mL for more than six months after the treatment. |
| **Expected Research Period** | From the time point when this research was approved by an international review board (IRB) to April 30, 2015 |
| **Phase** | Phase 4 |
| **Study Design** | Multicenter prospective, randomized open label clinical trial |
| **Method** | The CHB patients (both HBeAg-positive and – negative) who have at least 6 months undetectable HBV DNA (serum HBV DNA ≤ 20 IU/mL) after lamivudine plus adefovir combination therapy will be randomized in a 1:2 to treatment arm A or B.  All subjects will be treated for 96 weeks with one of treatment A and B according to randomization |
| **Number of Subjects Planned** | 171 subjects |
| **Major inclusion criteria** | Chronic hepatitis B patients who were found to be resistant to Lamivudine while treated with Lamivudine, underwent combination therapy of Lamivudine and Adefovir, and maintained HBV DNA negativity(≤20 IU/mL) for more than six months |
| **Exclusion criteria** | 1) Patients with decompensated liver cirrhosis or Child-Pugh class B or C  2) Patients with hepatitis C virus (HCV) or human immunodeficiency virus (HIV)  3) Patients with liver cell carcinoma  4) Patients whose alanine aminotransferase (ALT) level is twice the normal value  5) Patients whose blood creatinine level is 2.0mg/dL or above or who undergo dialysis  6) Pregnant or lactating women  7) Women who have a plan for pregnancy within the three coming years  8) Patients who have severe concomitant diseases—all kinds of cancers, severe cardiovascular diseases, renal inadequacy, peritonitis, and sepsis  9) Those who have no capabilities to understand and sign an informed consent |
| **Visit schedule** | 0, 1, 3, 6, 9, 12, 15, 18, 21, 24 month |
| **Statistical analysis** | A chi-square test was used for categorical variables, and the Fisher’s exact test was used when appropriate. Continuous variables with skewed distribution were analyzed using the Mann-Whitney test. P<0.05 was considered statistically significant. |
| **Requester** |  |

**Study protocol**

1. Study Title

Randomized Trial of Tenofovir versus Lamivudine plus Adefovir in Lamivudine plus Adefovir Treated Lamivudine-resistant chronic hepatitis B patients with Undetectable Hepatitis B Virus DNA

1. Location and address
   Keimyung University Dongsan Medical Center

/ 56 Dalseong-ro Jung-gu Daegu, Republic of Korea

1. Investigators

Principal Investigator: Byoung Kuk Jang, M.D Keimyung University Dongsan Medical Center

Investigator: Jae Seok Hwang, M.D Keimyung University Dongsan Medical Center

Investigator: Woo Jin Chung, M.D Keimyung University Dongsan Medical Center

Investigator: Boram Min, M.D Keimyung University Dongsan Medical Center

* Collaborators

Kyungpook National University

Daegu Catholic University Medical Center

DongGuk University

Pusan National University Hospital

Yeungnam University Hospital

1. Sponsor

Gilead science.

1. Study Purpose and Background

Recently, long-term medication of antiviral agents and their resulting resistance expression have been the most serious cause of failure to treat chronic hepatitis B in Korea. Expression of resistance to agents results in proliferation of mutations, triggering virus breakthrough and possibly aggravating chronic hepatitis B. In particular, the annual resistance rate to lamivudine currently widely being used in Korea amounts to about 15 to 20 percents and the rate is expected to reach 70 to 80 percent in four to five years [1]. The guidelines by the American Association for the Study of Liver Disease (AASLD) and the European Association for the Study of the Liver (EASL) [2, 3] recommend a combination therapy where Adefovir or Tenofovir are added in order to treat resistance to Lamivudine or a method to administer combined products of Tenofovir and Emtricitabine. In Korea, however, in case of combined prescription of Lamivudine and Adefovir, only one of them is covered by the health insurance and therefore many patients find it difficult to continue treatment due to their economic conditions.

Tenofovir that has been developed most recently and will be placed on sale sooner or later in Korea has strong antivirual effects, causes little or no emergence of resistant viruses, and is known to have lower neprotoxicity than Adefovir [4]. In particular, Tenofovir was reported to have effective and sustaining antiviral effects in patients who had been exposed to other antiviral agents as well as those who received initial treatment. Bommel et al. [5] reported that when 131 patients with either Lamivudine or Adefovir resistance were treated only with Tenofovir for an average period of 23 months, 79 percent of them achieved hepatitis B virus (HBV) DNA <400 copies/mL and, in particular, in case of those with Lamivudine resistance, all of them reached the same level. Furthermore, when patients with Adefovir resistance were treated merely with Tenofovir, only 52 percent were HBV DNA negative, which was low, and their levels had significant relation with the levels prior to the treatment. This shows that patients only with Lamivudine resistance can be treated merely with Tenofovir without a combination therapy and when they have low levels of HBV DNA, treatment is relatively effective despite their resistance to Adefovir. Moreover, according to Berg et al., 46 percent and 64 percent of patients with Lamivudine resistance who incompletely responded to monotherapy of Adefovir or combination therapy of Lamivudine and Adefovir which were applied for 24 weeks (HbeAg (+) :HBV DNA > 10^5^ copies/ml, HbeAg(-) : HBV DNA >10^4^ copies/mL) were HBV DNA negative when they underwent combination therapy of Lamivudine and Tenofovir for 48 weeks and 96 weeks, respectively [6]. Therefore, it is considered that when treatment for patients with Lamivudine resistance who have already received combination therapy of Lamivudine and Adefovir, whose HBV DNA negativity was well maintained, and who were not Adefovir resistant is converted to monotherapy of Tenofovir, they are likely to maintain their virus response. However, there are no relevant data. Accordingly, this study intends to examine effect of monotherapy of Tenofovir on such patients, factors that affect its treatment effect, and its side effects, thereby providing a new guideline to treat patients with Lamivudine resistance.

1. Intervention drug name and information

1-1.Lamivudine

1) Generic/Brand name: Lamivudine/Zeffix®

2) Ingredients Amount of drug / ingredient: 100mg in one tablet

3) Formulation: Film-coated tablet

4) Usage: Oral administration once a day

5) Pharmaceutical Company / Manufacturer: GlaxoSmithKline

1-2. Adefovir

1) Product name: Adefovir / Hepsera®

2) Ingredients Amount of drug / ingredient: 10mg in one tablet

3) Formulation: Film-coated tablet

4) Usage: Oral administration once a day

5) Pharmaceutical Company / Manufacturer: GlaxoSmithKline

2. Tenofovir

1) Chemical Name / Product Name: Tenoforvir / Viread®

2) Ingredients Amount of drug / ingredient: 300mg in one tablet

3) Formulation: Film-coated tablet

4) Usage: Oral administration once a day

5) Pharmaceutical Company / Manufacturer: Gilead

1. Target subject

The CHB patients (both HBeAg-positive and - negative) who have at least 6 months undetectable HBV DNA (serum HBV DNA ≤ 20 IU/mL) after lamivudine plus adefovir combination therapy

1. Expected Research Period
   From the time point when this research was approved by an international review board (IRB) to May 31, 2015
2. Methods
   1. Outline

1. We converted the treatment for chronic hepatitis B patients with Lamivudine resistance who were treated with combination therapy of Lamivudine and Adefovir, whose HBV DNA negativity was maintained for more than six months during the treatment, and who did not have Adefovir resistance to monotherapy of Tenofovir and examined their virus reactivation rate and types of drug resistance.

2. The criteria of virus reactivation is defined as HBV DNA levels >40 IU/mL at 2 consecutive time-points or persistent HBV DNA levels of 20–40 IU/mL at 3 consecutive time-points

3. In case of a virus reactivation, treatment is made of adding Lamivudine to Tenofovir.

4. Primary end point : when a virus reactivation occurs

5. Secondary end point : when a drug resistant virus appears (biochemical breakthrough)

- 1. Standard for selecting and excluding subjects

* Standard for selecting subjects

1. Male and female patients aged 18 or older

2. Chronic hepatitis B patients who were found to be resistant to Lamivudine while treated with Lamivudine, underwent combination therapy of Lamivudine and Adefovir, and maintained HBV DNA negativity or HBV DNA ≤ 20 IU/mL for more than six months

3. Chronic hepatitis patients or patients with compensated liver cirrhosis (Child-Pugh class A)

* Standard for excluding subjects

1. Patients with decompensated liver cirrhosis or Child-Pugh class B or C

2. Patients with hepatitis C virus (HCV) or human immunodeficiency virus (HIV)

3. Patients with liver cell carcinoma

4. Patients whose alanine aminotransferase (ALT) level is twice the normal value

5. Patients whose blood creatinine level is 2.0mg/dL or above or who undergo dialysis

6. Pregnant or lactating women

7. Women who have a plan for pregnancy within the three coming years

8. Patients who have severe concomitant diseases—all kinds of cancers, severe cardiovascular diseases, renal inadequacy, peritonitis, and sepsis

9. Those who have no capabilities to understand and sign an informed consent

- 1. Number of Subjects Planned
     171 subjects, randomized in a 1:2 ratio to maintain lamivudine plus adefovir treatment or switch to tenofovir 300mg monotherapy. With respect to the primary efficacy endpoint, sample sizes of 57 lamivudien plus adefovir maintain group and 114 tenofovir monotherapy group provide at least 80% power to detect a difference of less than 6% between the two groups, based on a Chi-squre or Fisher's exact test with a significance level of 0.05.

1:2

Group A : N/(1-0.2)=N/0.9=50.49/0.9=57

Group B : 2N=114 Total 171

| **alpha** | **beta** | **δ** | **p_c -_ p_t_** | **p_c_** | **p_t_** | **lambda** | **sample size** |
| --- | --- | --- | --- | --- | --- | --- | --- |
| **0.050** | **0.200** | **0.060** | **0.000** | **0.020** | **0.020** | **2.000** | **50.491** |

Assuming a dropout rate of 10 percent during clinical trial period (2 years), the number of patients required overall is estimated to be 171.

- 1. Randomization

At the beginning of the study, lamivudine and adefovir combined administration and Tenofovir alone were randomly assigned to open-label studies. Block randomization is used to assign drugs. All patients undergo random assignment at the core center (CRA) and are assigned patients in a competitive enrollment manner.

- 1. Observation items, observational testing methods, and clinical testing items

1. Birth date, gender, and age

2. Subjects’ liver functions, prothrombin time (PT), and HBV DNA are measured prior to

the treatment and one month and three months after the treatment and every three months thereafter until 24 months after the treatment

3. Alpha-fetoprotein (AFP) measurement, ultrasonography (USG) or computed

tomography (CT) are performed pursuant to the guideline by the Korean Association for the Study of the Liver

4. The Child-Pugh (CP) score of patients with liver cirrhosis is measured every three

months

5. A virus resistance test is performed on the subjects prior to the treatment, one year after the treatment, and two years after the treatment

6. The subjects are tested for Hepatitis B e Antigen/Antibody (HbeAg/Ab) prior to the treatment and every six months thereafter

7. The subjects are tested for Hepatitis B s antigen/ antibody (HBsAg/Ab) prior to the treatment and every one year thereafter

8. Keep a portion of the blood used for the test and use it for unknown mutation tests

- 1. Compliance testing

1. Antiviral agents are prescribed in hospital and whether the subjects received them should be confirmed

2. On a follow-up hospital visit, the subjects were required to bring residual drugs to check their amount, which should be written in the case report form (CRF)

- 1. Effect evaluation variables, evaluation methods and interpretation methods

Supplement (1)

- 1. Safety evaluation standards, evaluation methods and reporting methods

Supplement (1)

- 1. Criteria for suspension or withdrawal of clinical research

Standard dropout criteria

1) If the subject withdraws his / her consent to participate in the examination

2) Serious side effects

Statistical analysis

We will perform technical statistical analysis on all variables to be analyzed. Data for categorical variables are expressed in frequency and incidence. Data for continuous variables are expressed as mean and standard deviation, but if they are not normal distribution, they will be indicated as intermediate value and interquartile range. Comparisons between subgroups will be analyzed using t-test or χ-test. Multiple regression analysis will provide independent predictors of virus reactivation and resistance to viral emergence. The p-value is expected to be statistically significant less than 0.05.

1. Consideration of the safety of the subjects (stopping the test, handling serious side effects, etc.)

You may be restricted from participation in this study without your consent if:

A. Do not follow the instructions of your research doctor

B. Significant illnesses not related to research participation

C. Your research doctor decides that research is not the best way for you.

D. If you are pregnant, intend to be pregnant, or are breastfeeding during the study period

By participating in this trial, there are no adverse side effects from the medical side. However, if physical injury or injury directly related to participation in this trial occurs in the Gulf, the researcher will receive appropriate treatment at the institution hospital to which it belongs. If the subject is experiencing any adverse events related to the test, or if additional information is needed about your rights and responsibilities when taking the test and taking the test, please contact your doctor at any time during the trial.

1. Study schedule

| Study period : Approved by an IRB ~ April 30, 2015 | | | | | | | | | | | | | | | |
| --- | --- | --- | --- | --- | --- | --- | --- | --- | --- | --- | --- | --- | --- | --- | --- |
| Month | 10 | 12 | 8 | 12 | 4 | 8 | 12 | 4 | 8 | 12 | 2 | 6 | 8 | 10 | 12 |
| 1. IRB Review 2. Search for medical records 3. Enter the data 4. Results Analysis | O | O  O  O | O  O | O  O | O  O | O  O | O  O | O  O | O  O | O  O | O  O | O  O | O | O | O |

1. Matters necessary for ensuring the ethical and scientific quality of other clinical trials
2. Refrence

1. Locarnini S. Molecular virology and the development of resistant mutants: implications for therapy. Semin Liver Dis. 2005;25 Suppl 1:9-19. Epub 2005/08/17. doi: 10.1055/s-2005-915645. PubMed PMID: 16103977.

2. Lok AS, McMahon BJ. Chronic hepatitis B: update 2009. Hepatology. 2009;50(3):661-2. Epub 2009/08/29. doi: 10.1002/hep.23190. PubMed PMID: 19714720.

3. EASL Clinical Practice Guidelines: management of chronic hepatitis B. J Hepatol. 2009;50(2):227-42. Epub 2008/12/05. doi: 10.1016/j.jhep.2008.10.001. PubMed PMID: 19054588.

4. Heathcote EJ, Marcellin P, Buti M, Gane E, De Man RA, Krastev Z, et al. Three-year efficacy and safety of tenofovir disoproxil fumarate treatment for chronic hepatitis B. Gastroenterology. 2011;140(1):132-43. Epub 2010/10/20. doi: 10.1053/j.gastro.2010.10.011. PubMed PMID: 20955704.

5. van Bommel F, de Man RA, Wedemeyer H, Deterding K, Petersen J, Buggisch P, et al. Long-term efficacy of tenofovir monotherapy for hepatitis B virus-monoinfected patients after failure of nucleoside/nucleotide analogues. Hepatology. 2010;51(1):73-80. Epub 2009/12/10. doi: 10.1002/hep.23246. PubMed PMID: 19998272.

6. Berg T, Marcellin P, Zoulim F, Moller B, Trinh H, Chan S, et al. Tenofovir is effective alone or with emtricitabine in adefovir-treated patients with chronic-hepatitis B virus infection. Gastroenterology. 2010;139(4):1207-17. Epub 2010/07/06. doi: 10.1053/j.gastro.2010.06.053. PubMed PMID: 20600025.

1. Consent and explanation of the subject

Supplement (2)

1. Covenant on Damage Compensation (including directors' signature)

By participating in this trial, there are no adverse side effects from the medical side. However, if physical injury or injury directly related to participation in this trial occurs, the subject will receive appropriate treatment at the institution hospital to which the researcher belongs. Study subjects should contact their doctor at any time during the trial if adverse reactions to the study occur, or if additional information is needed about your rights and responsibilities when taking the test

1. Case Report Form

Supplement (1)
